# School Nurse Confidence with Diabetes Devices in Relation to Diabetes Knowledge and Prior Training: A Study of Convergent Validity

**DOI:** 10.1155/2023/2162900

**Published:** 2023-02-24

**Authors:** Christine A. March, Amber Hill, Traci M. Kazmerski, Linda Siminerio, Galen Switzer, Elizabeth Miller, Ingrid Libman

**Affiliations:** ^1^Department of Pediatrics, University of Pittsburgh, Pittsburgh, PA, USA; ^2^Division of Pediatric Endocrinology, UPMC Children's Hospital of Pittsburgh, Pittsburgh, PA, USA; ^3^University of Pittsburgh School of Medicine, Pittsburgh, PA, USA; ^4^Department of Pediatrics, Michigan Medicine, Ann Arbor, MI, USA; ^5^Division of Adolescent and Young Adult Medicine, UPMC Children's Hospital of Pittsburgh, Pittsburgh, PA, USA; ^6^Department of Medicine, University of Pittsburgh, Pittsburgh, PA, USA; ^7^Department of Psychiatry, University of Pittsburgh, Pittsburgh, PA, USA; ^8^Clinical and Translational Science Institute, University of Pittsburgh, Pittsburgh, PA, USA

## Abstract

**Objective:**

The diabetes device confidence scale (DDCS) is a new scale designed to evaluate school nurse confidence with diabetes devices. We hypothesized that the DDCS score would be associated with related constructs of school nurse diabetes knowledge, experience, and training. *Research Design and Methods*. In a cross-sectional study, we coadministered the DDCS and diabetes knowledge test 2 (DKT2) questionnaires to school nurses in Pennsylvania. We summarized DDCS scores (range 1–5) descriptively. We evaluated the relationship between the DKT2 percent score and DDCS mean score with the Spearman correlation coefficient. Simple linear regression examined school nurse characteristics as predictors of DDCS score.

**Results:**

A total of 271 completed surveys were received. The mean DDCS score was 3.16 ± 0.94, indicating moderate confidence with devices overall. School nurses frequently reported low confidence in items representing specific skills, including suspending insulin delivery (40%), giving a manual bolus (42%), knowing when to calibrate a continuous glucose monitor (48%), changing an insulin pump site (54%), and setting a temporary basal rate (58%). The mean DKT2 score was 89.5 ± 0.1%, which was weakly but not significantly correlated with the DDCS score (*r* = 0.12, *p*=0.06). Formal device training (*p* < 0.001), assisting ≥5 students with diabetes devices in the past 5 years (*p* < 0.01), and a student caseload between 1000 and 1500 students (*p* < 0.001) were associated with higher mean DDCS score.

**Conclusions:**

DDCS score is related to prior training and experience, providing evidence for the scale's convergent validity. The DDCS may be a useful tool for assessing school nurse readiness to use devices and identify areas to enhance knowledge and practical skills.

## 1. Introduction

Children with type 1 diabetes increasingly rely on diabetes devices, including insulin pumps, continuous glucose monitors (CGM), and sensor-integrated systems [1]. These devices, particularly when able to automate some aspects of insulin delivery, may improve glycemic control [2–4] and patient- and family-reported outcomes [5]. To successfully utilize this technology and attain the maximum benefit, leading organizations in pediatric diabetes recommend structured diabetes education for patients and families both with device initiation and their ongoing use [6, 7]. Equally important are guidelines for other caregivers, including school health staff and specifically school nurses, who also have frequent contact with children using diabetes devices [8].

School nurses are often the frontline for diabetes management in the school setting. Until they develop independent care skills, children with type 1 diabetes rely upon school health staff for assistance [9]. The Association for Diabetes Care and Education Specialists has recommended specific competencies for school nurses with devices, which includes supporting students through an individualized care plan [10]. School nurses agree that they need to be familiar with all aspects of devices [11]. Yet, different factors may affect school nurses' support of device use in school [12]. Some may be external, such as school policies related to cell phone access, students' abilities for self-management, and family support. However, internal factors are also important, including the school nurses' personal diabetes knowledge, confidence in using devices, and past experiences in their nursing and school nursing career [12].

Our prior research has examined the relationships between some of these factors for school nurses in the context of modern diabetes treatments. The diabetes device confidence scale (DDCS) was designed to evaluate school nurse confidence in performing practical skills with diabetes devices [13]. The DDCS demonstrated high content validity and internal consistency reliability with a unidimensional factor structure. The DDCS score was moderately positively correlated with school nurses' reported confidence in their professional skills generally, suggesting that the scale provides related, yet distinct, information in a particular area of diabetes management. The DDCS added to the limited existing literature on investigator-designed scales used to measure school nurse knowledge or confidence with general diabetes care and education in the context of a specific intervention [14–17].

The objectives of this secondary analysis were to further add to the convergent validity evidence of the DDCS by assessing whether confidence is associated with other, theoretically related constructs. We examined the associations between the DDCS and other internal factors, including school nurse diabetes knowledge, experience, and training. Given the demonstrated construct validity of the DDCS [13], we hypothesized that device confidence and diabetes knowledge would be related but not equivalently constructs, and device confidence would be associated with formal training and greater experience assisting children with type 1 diabetes who use devices.

## 2. Research Design and Methods

We examined school nurses' perceived confidence with diabetes devices in a statewide sample of school nurses through a cross-sectional survey using the DDCS. This study was a secondary analysis of data collected for the original psychometric testing of the DDCS. Full details regarding the design, distribution, and prior validity assessments for the DDCS are published elsewhere [13]. This study was deemed exempt by the University of Pittsburgh Institutional Review Board (PRO #20030097).

### 2.1. Questionnaires

The DDCS is a 25-item scale composed of questions regarding specific skills using diabetes devices, including insulin pumps, continuous glucose monitors, and sensor-integrated insulin pumps. School nurses self-assess their confidence with these skills when using diabetes devices on a 5-point Likert type scale from “not at all confident” to “extremely confident.” Designed by a collaborative team of diabetes providers, school nurses, and parents of youth with diabetes, the scale has undergone various assessments for face, content, structural, and convergent validity as well as internal consistency reliability [13]. The DDCS was found to have a single-factor structure, high reliability (alpha = 0.97), and a significant, though moderate, correlation with participants' general confidence in their nursing skills (*β* = 0.57, 95% CI = 0.5–0.69).

In addition to the DDCS, invited participants were asked background questions about their prior experience working with students who use diabetes devices, prior training with devices, personal demographics, and characteristics of their school employment. Prior training could include formal training through a health center or other mechanisms or informal methods through device representatives, parents, other school nurses, or online. We theorized that diabetes knowledge may be a related construct. To assess for convergent validity between device confidence and diabetes knowledge, we coadministered the diabetes knowledge test 2 (DKT2), a validated, multiple-choice test of diabetes knowledge which had previously been adapted for school personnel [18–20].

### 2.2. Distribution

Subjects were school nurses in Pennsylvania employed in grades K-12 in either public or private schools. School nurses received an invitation to participate in the survey through an e-mail distribution list for the Pennsylvania Association for School Nurses And Practitioners (PASNAP), the statewide branch for the National Association for School Nurses (NASN). PASNAP has approximately 900 active members. The survey was administered online through the University of Pittsburgh Qualtrics system with an initial recruitment script and five reminder notifications. School nurses who completed the survey were eligible to win a gift card as an incentive. Data were collected from October–December 2020.

### 2.3. Analysis

We tabulated descriptive statistics for school nurse-reported background characteristics, including means with standard deviations and counts with proportions. DDCS responses are presented as a mean and standard deviation for each individual item and for the whole scale (25 items). The DKT2 was scored as the percentage of questions which were answered correctly. To assess for convergent validity, we examined the relationship between diabetes knowledge (measured by the DKT2) and device confidence (measured by the DDCS) using Spearman's rank correlation coefficient. We evaluated for predictors of the DDCS score using selected background characteristics, including age, years of school nursing experience, student caseload, prior formal training, and the number of students with type 1 diabetes who use devices in the past 5 years, using simple linear regression. These predictors were selected based upon prior qualitative studies in which school nurses self-identified factors which may affect their comfort with diabetes devices [11, 14, 21, 22]. Significance was considered with a *p* < 0.05. All analyses were conducted in STATA SE v17.0.

## 3. Results

A total of 310 school nurses completed the online survey; 39 were excluded as the respondents did not complete the DDCS, leaving a final sample of 271. Background characteristics for the study sample have previously been described and are included in Table 1 [13]. Approximately half of the surveyed school nurses had at least 10 years of experience in that role and the majority (87%) worked in public schools only. The school nurses covered a variety of grades in different geographical settings. Student-to-school nurse ratio (caseload) also varied, with most reporting 500–1500 students.

Regarding their experiences with diabetes care, the surveyed school nurses reported a high degree of familiarity with devices (Table 2), with >90% previously working with a student who used a CGM or insulin pump. Approximately half (54%) had assisted at least five students with diabetes who use devices in the past five years. A little over half (52%) reported prior formal training with diabetes devices through a medical center or other organization; many also reported utilizing informal approaches to training, including learning from parents (68%), other school nurses (48%), or web searches (49%).

The average score on the DDCS was 3.16 ± 0.94, indicating a moderate degree of confidence with diabetes devices (Table 3). Mean scores on individual items ranged from 2.2 ± 1.3 (“I can operate a student's sensor-integrated pump if needed”) to 3.9 ± 1.1 (“I can help a teacher/other school staff understand what to do if a student's CGM alarms in class”). There was a great degree of variability in school nurse responses across items indicating different skill levels. A large proportion of school nurses indicated low confidence (choice of 1 or 2 on the Likert scale) in very specific skills, including checking insulin pump settings (38%), giving a manual bolus in the pump (42%), suspending insulin delivery (40%), setting a temporary basal rate on an insulin pump (58%), changing an insulin pump site (54%), operating a sensor-integrated pump (64%), or knowing when to calibrate a CGM (48%).

In contrast, school nurses reported a high degree of confidence (choice of 4 or 5 on the Likert scale) with skills mostly related to oversight, education, and counseling. These included recognizing a pump site failure (58%), assessing a student's knowledge about their insulin pump (64%) or CGM (59%), interpreting trend arrows on a CGM tracing (51%), recognizing when to test with a glucometer rather than use CGM (73%), helping a teacher understand what to do if a student's CGM alarms in class (70%), learning how to use a new device (66%), setting expectations for device use in school (65%), and finding resources for school staff (63%).

The mean score on the DKT2 was 89.5 ± 0.1% (range 57–100%). The DKT2 percentage score was only weakly correlated with the mean DDCS score (*r* = 0.12, *p*=0.06). In simple linear regression, formal device training (*p* < 0.001), assisting at least five students who use diabetes devices in the past five years (*p*=0.002), and a student caseload between 1000 and 1500 students (*p*=0.004) were associated with higher mean DDCS score (Table 4). Age, years of school nursing experience, and presence of an advanced degree were not significantly associated with the mean DDCS score.

## 4. Discussion

The DDCS is a new tool designed to assess school-nurse confidence with diabetes devices which has undergone initial psychometric testing. In this secondary analysis examining DDCS scores, this study provides more insight into the specific skills in which school nurses feel more or less confident. Within this population of school nurses with a high degree of prior exposure to diabetes devices, the reported overall confidence in assisting students was moderate on a Likert scale.

When examining individual items on the DDCS, school nurse confidence was higher for skills related to oversight, counseling, and teaching others, likely reflecting expected responsibilities for this role in assisting children with type 1 diabetes or other chronic diseases of childhood [23]. School nurses reported lower confidence with many concrete skills with devices, such as administering a manual bolus or suspending insulin delivery on a pump. Possible explanations include infrequent exposure to the devices in question, inadequate training, or student populations which tend to be more independent in their diabetes management, which would limit the opportunities for school nurses to work directly with the devices [11].

The item with the lowest reported confidence concerned sensor-integrated insulin pumps, likely due to less experience with this technology (reported by only one-third of school nurses surveyed). This survey was distributed in 2020 at a time when automated insulin delivery systems were just becoming available for younger pediatric patients; the MiniMed Medtronic 670G™ pump with the Guardian Sensor™ was approved down to age 7 years in 2018 and the Tandem X2 pump™ with the Control IQ™ algorithm was approved and lowered to 6 years of age in the summer of 2020. As sensor-integrated technology becomes more prevalent among youth using insulin pumps, school nurse confidence with these systems is likely to improve over time. Though automated systems may also reduce the need for frequent school nurse intervention, school nurses should be comfortable with the basic mechanics of this technology should any issues arise during the school day and to assist younger children who are not yet independent in their management.

The findings of this study also provide evidence of the convergent validity of the DDCS. One of the theorized uses for this scale is to assess school nurse readiness to work with diabetes devices. In social cognitive theory, capability (knowledge and skills) and self-efficacy (confidence) are separate, though related, constructs that both contribute to behavior [24, 25]. We hypothesized that diabetes knowledge and device confidence would be related constructs. Our findings indicated a weak correlation, which adds to the validity that the DDCS measures a related, though distinct concept from diabetes knowledge. However, the relationship between the DDCS and diabetes knowledge may be affected to some degree by the content of the DKT2. Though the DKT2 has been validated in different populations and used with school personnel specifically [18–20], the items cover basic diabetes management and have not been adequately updated to address knowledge with diabetes technology. After completion of this study, a recently published scale which assesses more modern aspects of diabetes knowledge may prove more useful for practical purposes and should be tested in relation to the DDCS [26, 27].

In addition to diabetes knowledge, we found that past formal device training and experiences in working with students who use devices were significant predictors of device confidence on the DDCS, whereas years of experience in school nursing, having an advanced degree, and age were not. This contributes to overall convergent validity as confidence with devices may be less related to general school nursing experience or recent completion of undergraduate or graduate nursing programs and may be more related to diabetes-specific education or experiences [11, 14, 21]. Our findings highlight that formal training, whether through a diabetes center or other organization (e.g., NASN) can predict higher mean scores on DDCS. However, access to training tailored to school nurses remains a challenge [11] as options for centralized programs are limited and may rely on access via an organizational membership or diabetes center with the resources to host a training [16, 28]. Consistent practice guidelines and educational resources may help to standardize expectations for device use in school [29–31].

Our findings also indicated that the number of students using devices in the past five years was a significant predictor of confidence on the DDCS. In qualitative studies, school nurses have indeed identified real-world experience as one of the most important methods to increase their comfort with diabetes management, including devices [11, 14, 21]. This is further supported by the relationship between student caseload and DDCS score. A medium caseload (1000–1500 students) was a significant predictor of higher DDCS scores, which may also indicate greater exposure to students with diabetes using devices. Though hands-on experience is valuable on an individual level, it does not alleviate the need for device-specific education given national challenges with nursing shortages and staff turnover [32] and continued developments in new devices.

Regardless, this study does have limitations. This was a secondary analysis of data collected for the initial psychometric testing of the DDCS, which included additional items that were subsequently removed. Therefore, the relationships between confidence, measured by the DDCS score, with other variables should be further examined in a different sample using the condensed, 25-item scale only. Our findings do not imply that device confidence or diabetes knowledge translates to the actual performance of these skills. Using an objective evaluation of school nurse abilities with devices, such as direct observation of school nurses completing certain tasks, in comparison to perceived knowledge or confidence may identify deficiencies [17]. An additional limitation of this study is the self-selection to participate in completing the survey, as school nurses with low confidence or limited prior experience with devices may have chosen not to participate. In particular, fewer nurses participated from urban settings, which may be underresourced [32]. The timing of the survey, during the pandemic, may have affected school nurses' exposure to devices and, in turn, their answers, depending upon whether their schools were virtual. Lastly, we also did not survey other school caregivers, such as teachers or administrators, who may help fill the gap in diabetes management when school nurses are unavailable, depending upon state law.

Future work should examine the performance of the DDCS in other geographic settings and examine whether these relationships persist, which would further support its utility in clinical practice. The DDCS may be a useful adjunct to diabetes knowledge assessments for school districts to comprehensively evaluate school nurse readiness with diabetes devices for continuing education. Other potential applications of the DDCS include examining the effectiveness of interventions targeting school nurses or identifying gaps in confidence which can be used to develop and tailor educational programs. This study adds to the initial psychometric data [13] indicating that the DDCS measures a construct (confidence) distinct from diabetes knowledge which is related to prior training and experience with students who use devices. As devices are increasingly becoming the standard of care in pediatric type 1 diabetes management, ensuring school nurses are well-equipped to assist these students is essential to promote a healthy and safe learning environment.

## Figures and Tables

**Table 1:** Background characteristics for responding school nurses.

	*N* *=* 271
Demographic information	
White	259 (96)
Female	268 (99)
Age (years)	51.9 ± 9.1
School nursing background	
Years as a school nurse	
<5	51 (19)
5–10	79 (29)
11–20	95 (35)
>20	46 (17)
Advanced degree (masters, CRNP, or equivalent)	115 (42)
Certification in school nursing	238 (88)
Characteristics of current school employment	
Number of schools covered	
1	147 (54)
2 or more	123 (46)
Geographic region	
Urban	32 (12)
Suburban	141 (52)
Rural	98 (36)
School type	
Public	235 (87)
Private	3 (3)
Public and private	27 (10)
Grades covered	
Elementary (K-5)	147 (54)
Middle (6–8)	102 (38)
High (9–12)	99 (37)
Current caseload, students	
<500	44 (16)
501–1000	121 (45)
1001–1500	96 (35)
>1500	10 (4)

Notes: data displayed as *n* (%) or mean ± SD. Small amounts of missing data may lead to the *n* not totaling to 271.

**Table 2:** School nurse-reported experience with diabetes devices and prior training.

	*N* = 271
Number of children with diabetes devices cared for in school in the past 5 years	
<5 students	125 (46)
5–10 students	98 (36)
11–15 students	26 (10)
16–20 students	12 (5)
≥20 students	9 (3)
Prior exposure to devices	
Insulin pumps	257 (95)
Continuous glucose monitors	248 (92)
Sensor-augmented insulin pumps	90 (34)
Prior training with devices	
Learning from parents/guardians	184 (68)
Informal approach through web search	132 (49)
Learning from school nurses on the job	130 (48)
Formal training from a medical center	101 (37)
Formal training found independently	71 (26)
Training from a device rep	72 (27)
Any formal training	152 (52)

Notes: data displayed as *n* (%). Small amounts of missing data may lead to the *n* not totaling to 271. For prior training with devices, respondents were able to select more than one option.

**Table 3:** Mean score and likert scale responses for each DDCS item.

Item	DDCS score	Not at all confident	Somewhat confident	Moderately confident	Very confident	Extremely confident
	Mean ± SD	*N* (%)				
I can give a combined food and correction bolus using an insulin pump	3.2 ± 1.3	40 (15)	41 (15)	59 (22)	75 (28)	55 (20)
I can override an insulin pump to give a bolus (not using the pump's calculations)	2.8 ± 1.4	66 (24)	48 (18)	61 (23)	59 (22)	35 (13)
I can set a temporary basal rate on an insulin pump	2.4 ± 1.3	97 (36)	59 (22)	47 (17)	47 (17)	20 (8)
I can suspend insulin delivery on an insulin pump	2.9 ± 1.4	59 (22)	48 (18)	67 (25)	56 (20)	41 (15)
I can change an insulin pump site	2.5 ± 1.4	95 (35)	51 (19)	54 (20)	36 (13)	34 (13)
I can check insulin pump settings (basal rate, insulin to carb ratio, and correction factor)	3.0 ± 1.3	39 (15)	63 (23)	67 (25)	62 (23)	38 (14)
I can review the bolus history	3.2 ± 1.3	35 (13)	51 (19)	65 (24)	71 (26)	48 (18)
I can change a battery or charge the insulin pump	3.0 ± 1.4	52 (19)	41 (15)	66 (25)	65 (24)	46 (17)
I recognize when an insulin pump site has failed	3.6 ± 1.2	18 (7)	31 (12)	62 (23)	95 (35)	63 (23)
I understand what a sensor-integrated insulin pump is	2.6 ± 1.3	77 (28)	48 (18)	69 (26)	54 (20)	21 (8)
I can operate a student's sensor-integrated pump if needed	2.2 ± 1.3	112 (42)	60 (22)	45 (17)	39 (14)	14 (5)
I can adequately assess a student's knowledge about their insulin pump	3.7 ± 1.1	11 (4)	24 (9)	63 (23)	108 (40)	64 (24)
I can help a student manage their diabetes with a variety of insulin pumps	3.1 ± 1.3	37 (14)	53 (20)	60 (22)	84 (31)	36 (13)
I can operate a student's CGM if needed	3.0 ± 1.3	47 (17)	46 (17)	75 (28)	64 (24)	38 (14)
I know when to calibrate and when not to calibrate a CGM	2.6 ± 1.3	71 (26)	60 (22)	62 (23)	51 (19)	26 (10)
I can monitor the CGM tracing/graph when needed	2.9 ± 1.4	60 (22)	54 (20)	66 (24)	42 (16)	48 (18)
I can interpret trend arrows on a CGM tracing	3.4 ± 1.4	38 (14)	38 (14)	56 (21)	66 (24)	73 (27)
I understand what the different CGM alarms mean	3.1 ± 1.4	52 (19)	44 (16)	64 (24)	56 (21)	54 (20)
I recognize when to test a glucose using a meter for a student with a CGM	3.9 ± 1.1	16 (6)	15 (5)	43 (16)	92 (34)	105 (39)
I can adequately assess a student's knowledge about their CGM	3.6 ± 1.1	14 (5)	27 (10)	70 (26)	95 (35)	64 (24)
I can help a teacher/other school staff understand what to do if a student's CGM alarms in class	3.9 ± 1.1	16 (6)	26 (9)	40 (15)	89 (33)	100 (37)
I can help a student manage their diabetes with a variety of CGMs	3.2 ± 1.4	48 (18)	33 (12)	72 (27)	66 (24)	52 (19)
I can learn how to use a diabetes device that I am not familiar with	3.8 ± 0.9	3 (1)	20 (7)	70 (26)	106 (39)	72 (27)
I can set expectations for device use in school with parents	3.8 ± 1.0	8 (3)	17 (6)	69 (26)	95 (35)	82 (30)
I can easily find resources to teach school staff about diabetes devices	3.7 ± 1.0	5 (2)	31 (11)	64 (24)	99 (36)	27 (27)

**Table 4:** School nurse predictors of mean DDCS score using simple linear regression.

Characteristic	*B* value	95% CI	*p* value
Age	−0.010	(−0.02, 0.003)	0.13
Years as a school nurse			
<5	Ref	—	—
5–10	0.191	(−0.142, 0.525)	0.260
11–20	0.123	(−0.200, 0.445)	0.454
>20	0.163	(−0.214, 0.541)	0.395
Advanced degree	0.105	(−0.123, 0.333)	0.365
Caseload			
<500	Ref	—	—
500–999	0.282	(−0.041, 0.604)	0.087
1000–1500	0.493	(0.160, 0.826)	0.004
>1500	0.150	(−0.492, 0.792)	0.646
Formal device training	0.565	(0.349, 0.780)	<0.001
≥5 students with devices in past 5 years	0.361	(0.138, 0.584)	0.002

## Data Availability

All the data used in this research study can be found in this manuscript.
